# Successful adaptation of twinning concept for global neurosurgery collaborations—a validation study

**DOI:** 10.1007/s00701-024-06060-8

**Published:** 2024-04-11

**Authors:** Alba Corell, John N. Jabang, Job Manneh, Ebrima K. Manneh, Magnus Tisell, Christian Brandt, Tomas Majing, Camilla Smedberg, Charlotte Förars, Sarinah Rebucas, Pascal Goswell, Tove Ronold, Caroline Landén, Anders Engström, Pia Sorto, Enoch Uche, Daouda Wague, Youssoupha Sakho, Jimmy Sundblom

**Affiliations:** 1 Department of Neurosurgery, Sahlgrenska University Hospital, Blå Stråket 5, 41345 Gothenburg, Sweden; 2 Institute of Neuroscience and Physiology, Department of Clinical Neuroscience, University of Gothenburg, Sahlgrenska Academy, Gothenburg, Sweden; 3 Neurosurgical Unit, Department of Surgery, EFSTH Teaching Hospital, Banjul, The Gambia; 4 Neurosurgical Department, Lund University Hospital, Lund, Sweden; 5 Department of Perioperative Medicine and Intensive Care, Karolinska University Hospital, Stockholm, Sweden; 6 Department of Neurosurgery, Karolinska University Hospital, Stockholm, Sweden; 7 Department of Neurosurgery, Uppsala University Hospital, Uppsala, Sweden; 8 Department of Rehabilitation, Sahlgrenska University Hospital, Gothenburg, Sweden; 9 Department of Strategic Planning, Sahlgrenska University Hospital, Gothenburg, Sweden; 10 Department of Neurosurgery, University of Helsinki and Helsinki University Hospital, Helsinki, Finland; 11 Department of Neurosurgery, The University of Nigeria Teaching Hospital, Enugu, Nigeria; 12 Department of Neurosurgery, Fann University Teaching Hospital, Dakar, Senegal; 13 Department of Neurosurgery, Grand Yoff General Hospital, Dakar, Senegal

**Keywords:** Global neurosurgery, Sub-Saharan Africa, Neurosurgery, Brain tumors

## Abstract

**Introduction:**

Globally, many regions have an urgent, unmet need of neurosurgical care. A multi-step neurosurgical twinning technique, International Neurosurgical Twinning Modeled for Africa (INTIMA), was proved to be successful during a previous mission to Neurosurgical Unit, Enugu, Nigeria. The Swedish African Neurosurgical Collaboration (SANC) performed a developmental mission together with the local neurosurgical unit in The Gambia, adopting the INTIMA model.

**Methods:**

A multidisciplinary team visited for a 2-week collaborative mission at the Neurosurgical Department of the Edward Francis Small Teaching Hospital in Banjul, The Gambia. The mission followed the data of neurosurgical operations during and after the mission as well as about the operations 3 months prior to and after the mission was collected.

**Results:**

During the mission, a total of 22 operations was carried out, the most common being degenerative spinal conditions (*n* = 9). In the 3 months following the mission, 43 operations were performed compared to 24 during the 3 months leading up to the mission. The complexity of the performed procedures increased after the mission. An operating microscope (Möller-Wedel) was donated and installed and the neurosurgeons on site underwent training in microneurosurgery. The surgical nurses, nurses at the postoperative ward, and the physiotherapists underwent training. A biomedical engineer serviced multiple appliances and devices improving the patient care on site while training local technicians.

**Conclusion:**

This study validated the use of the INTIMA model previously described in a mission by Swedish African Neurosurgical Collaboration (SANC). The model is sustainable and produces notable results. The core strength of the model is in the multidisciplinary team securing all the aspects and steps of the neurosurgical care. Installation of an operating microscope opened for further microsurgical possibilities, improving the neurosurgical care in The Gambia.

## Introduction

Access to neurosurgical care is lacking in many regions globally, and the need for treatment of neurosurgical conditions is urgent. The neurosurgical units in West African countries and regions have different capabilities; infrastructure and the access to physicians to the neurosurgical training centers is inconsistent [2, 3]. Neurosurgery is a multidisciplinary specialty, where the collaboration between different occupations is essential to optimize the treatment of patients with neurosurgical conditions. Besides the operative treatment, the successful care for the neurosurgical patients includes postoperative care, neuro-intensive care, physiotherapy, and occupational therapy, among other services such as radiology, biomedical engineering, and laboratory services, to name a few.

To address these issues, global neurosurgery partnerships has been implemented and adopted across the world, with different strategies and models for collaboration. They have been instrumental in developing neurosurgery in low- and middle-income countries (LMIC), both delivering patient care but also creating sustainable long-term frameworks for neurosurgery [8].

Modern microneurosurgery as a discipline is resource-intense and establishing it in a new environment creates unique challenges. To address these challenges, the Swedish African Neurosurgical Collaboration (SANC) has created the International Neurosurgical Twinning Modeled for Africa (INTIMA), focusing on developing established but resource-challenged neurosurgical departments in the regions of need [5, 8].

The mission described here was initiated by an invitation from Edward Francis Small University Hospital (EFSTH) and builds upon the foundation laid by the previous SANC mission with the Neurosurgical Unit of the University of Nigeria Teaching Hospital [5]. The primary aim of this study was to validate the earlier multi-step neurosurgical twinning technique INTIMA, which has been found efficient [5]. As a secondary objective, we will present data in form of surgical volume prior to, during, and after the mission, in addition to the multidisciplinary efforts made during the mission.

## Methods

The EFSTH located in Banjul hosts the sole neurosurgical department of the country of The Gambia. The mission lasted between 2023–06-05 and 2023–06-16. The neurosurgical department is staffed with one neurosurgeon trained in microneurosurgery and two junior residents, and one operating room for neurosurgical patients. During the mission, the neurosurgical care was provided at the Edward Francis Small Teaching Hospital (EFSTH), Ndemban Branch, Bakau, The Gambia, with the access to two neurosurgical operating theaters.

The mission was performed on the basis of the previously described INTIMA model [5]. The phases of the model includes the Initial professional linkage (IPL) where professional friendships are created through meeting at courses and conferences. This is followed by Justification visit (JV) where an invitation is given to a neurosurgeon. In this case, the invitation came from Dr. John Jabang, EFSTH, Banjul, The Gambia. After the JV, the following phases include philanthropic travel (PT), followed by targeted benevolent donation (TBD) which for this mission the most important donation was the operating microscope (brand Möller-Wedel). Lastly, the phase of focus clinical partnerships (FCP) is initiated.

**Fig. 1:**
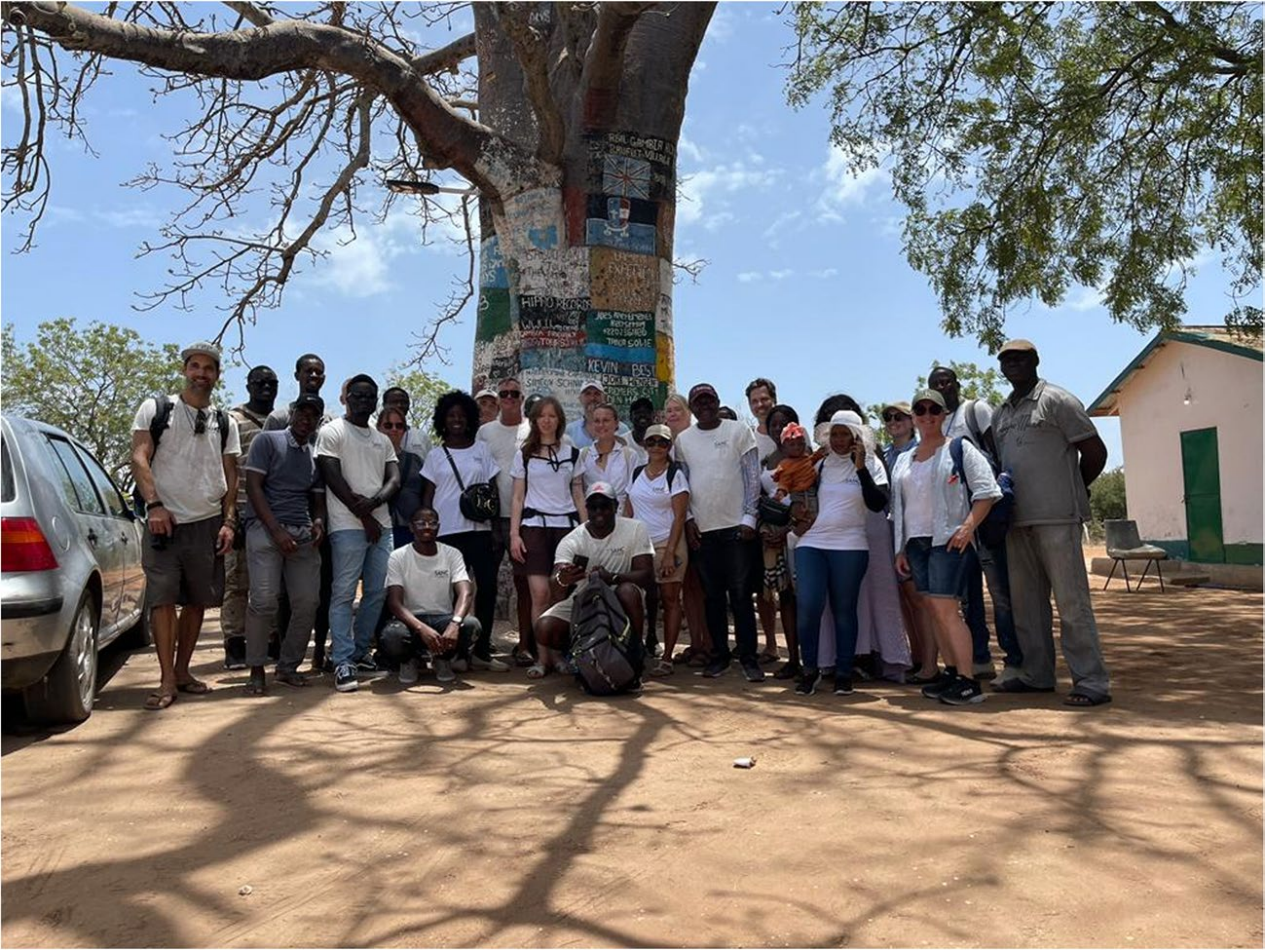
SANC and the local team at Edward Francis Small Teaching Hospital (EFSTH), Ndemban Branch, Bakau, The Gambia

The general mission aim was to create possibilities for microneurosurgery, in addition to develop the perioperative neurosurgical care. More detailed aims included the education of the surgical nurses in the operation room, and of the nurses in the perioperative and postoperative care units, as well as the education for patients with spinal cord injury. We delivered and installed an operating microscope (Fig. 1) and Mayfield head rest, Midas Rex™ high-speed drill, together with the other support with material and equipment. During the mission, SANC was included in a symposium on epilepsy and neurosurgery and to establish research collaboration.

**Fig. 2:**
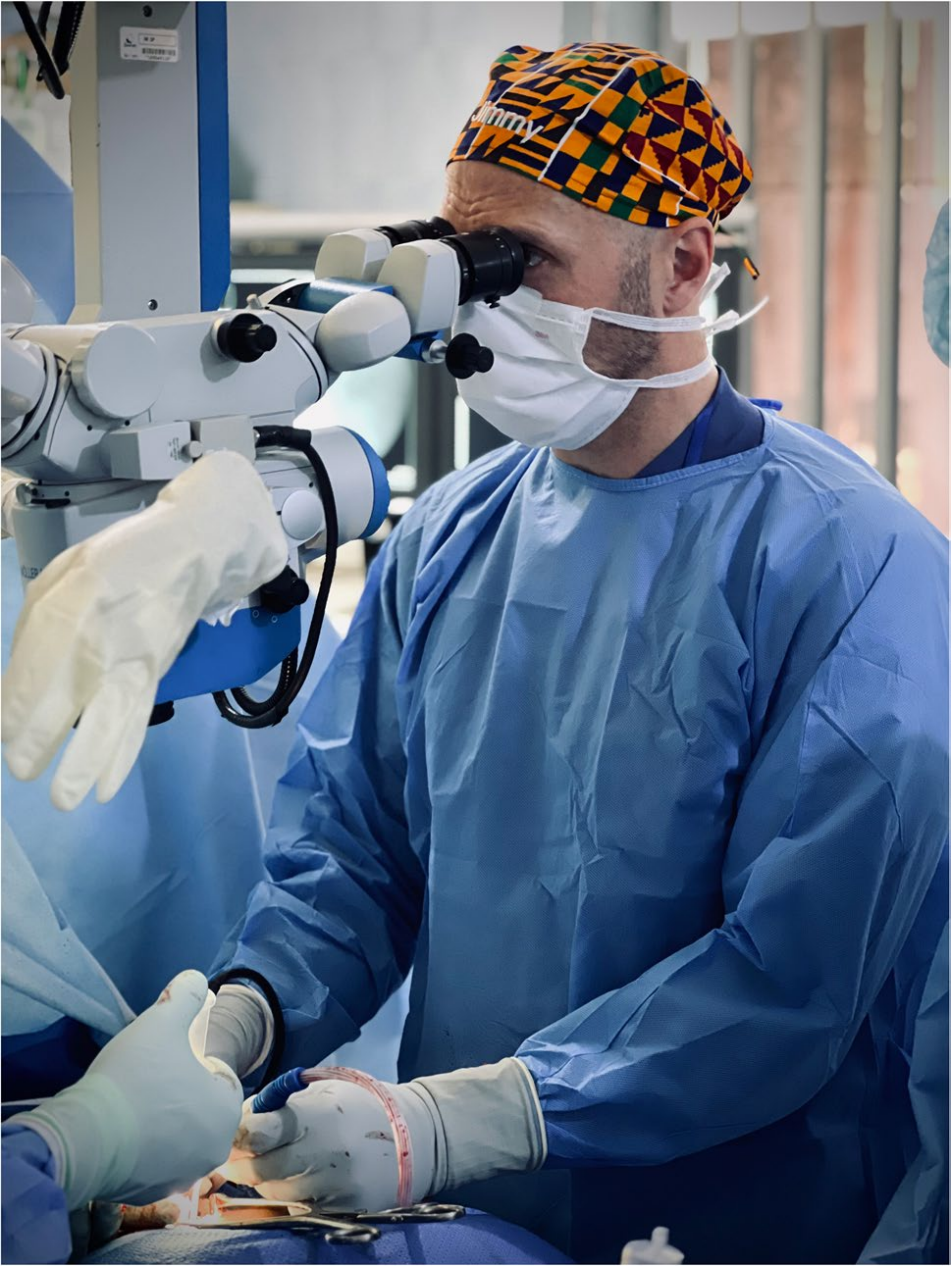
SANC team leader Dr. Jimmy Sundblom with the operating microscope Möller-Wedel

Before the mission, the SANC team held recurrent strategical meetings through which relationships between respective colleagues from the different occupational categories were established. The SANC team (led by JS, see Fig. 2) consisted of neurosurgeons (*n* = 5), neurosurgical surgical nurses (*n* = 2), assistant surgical nurse (*n* = 1), intensive care nurses with experience from neurointensive care (*n* = 2), occupational therapist (*n* = 1), biomedical engineer (*n* = 1), and neuroanesthesiologist (*n* = 1) (see Fig. 1). During part of the mission, a filmmaker was part of the team documenting this mission to further spread word of SANC.

### Time periods

This study comprises three time periods centered around the mission: (1) 3 months prior to the mission (P1, 2023–03-06 until 2023–06-04), (2) 10 days during the mission itself (P2, 2023–06-05 until 2023–06-16), and (3) the following three months after the mission ended (P3, 2023–06-17 until 2023–09-17). The data of the P1 period was collected retrospectively through operation logbooks at the neurosurgical department. As for the P2 and P3 periods, the data was collected prospectively.

### The complexity of performed surgeries

The complexity of operations performed was classified according to the British United Provident Association (BUPA) Schedule of procedures which score procedures on a five-grade scale. For this study we used surgeon’s category classified into minor, inter, major, major + , and complex major (CMO). CMO corresponds to a higher complexity.

### Statistical considerations

The descriptive statistics were produced using SPSS, version 29.0.1.1 (Chicago, IL, USA). No comparative analysis was performed.

## Results

### Case load

The number of cases and type of operation performed was recorded (see Table 1). During the 3 months prior to the mission (P1), a total of 24 procedures were performed in the one neurosurgical theatre. These included traumatic brain injuries (TBI), e.g., subdural hematomas and skull fractures (*n* = 10), hydrocephalus (*n* = 4), spinal trauma (*n* = 4), intracranial infections (*n* = 2), congenital conditions such as myelomeningocele or encephalocele (*n* = 2), one intracranial tumor, and one degenerative spinal condition.

**Table 1:** Overview of conditions treated over the time periods

Condition	Complexity level of the surgery	Time period			Total
		P1, 90 days before the mission	P2, 14 days during the mission	P3, 90 days after the mission	
Trauma TBI	MAJOR 1 – CMO 2	10	1	9	20
Intracranial abscess/empyema	CMO 3	2	1	4	7
Hydrocephalus	MAJOR 4	4	0	6	10
Spinal trauma	MAJOR + 4 – CMO 3	4	2	6	12
Congenital (MMC and encephalocele)	CMO 2	2	2	5	9
Tumor	CMO 4	1	5	3	9
Spinal degenerative	MAJOR + 4 – CMO 3	1	11	8	20
Vascular/stroke (endoscopic ventriculostomy)	MAJOR + 4	0	0	1	1
Unknown		0	0	1	1
Total		**24**	**22**	**43**	**89**

During the 12-day mission (P2), a total of 22 operations were performed, the most common being spinal degenerative conditions (including Potts disease) (*n* = 11), followed by extracranial tumor (*n* = 1) intracranial tumors (*n* = 3), intramedullary tumor (*n* = 1), spinal trauma (*n* = 2), congenital conditions (*n* = 2), intracranial infection (*n* = 1), and one TBI.

**Fig. 3:**
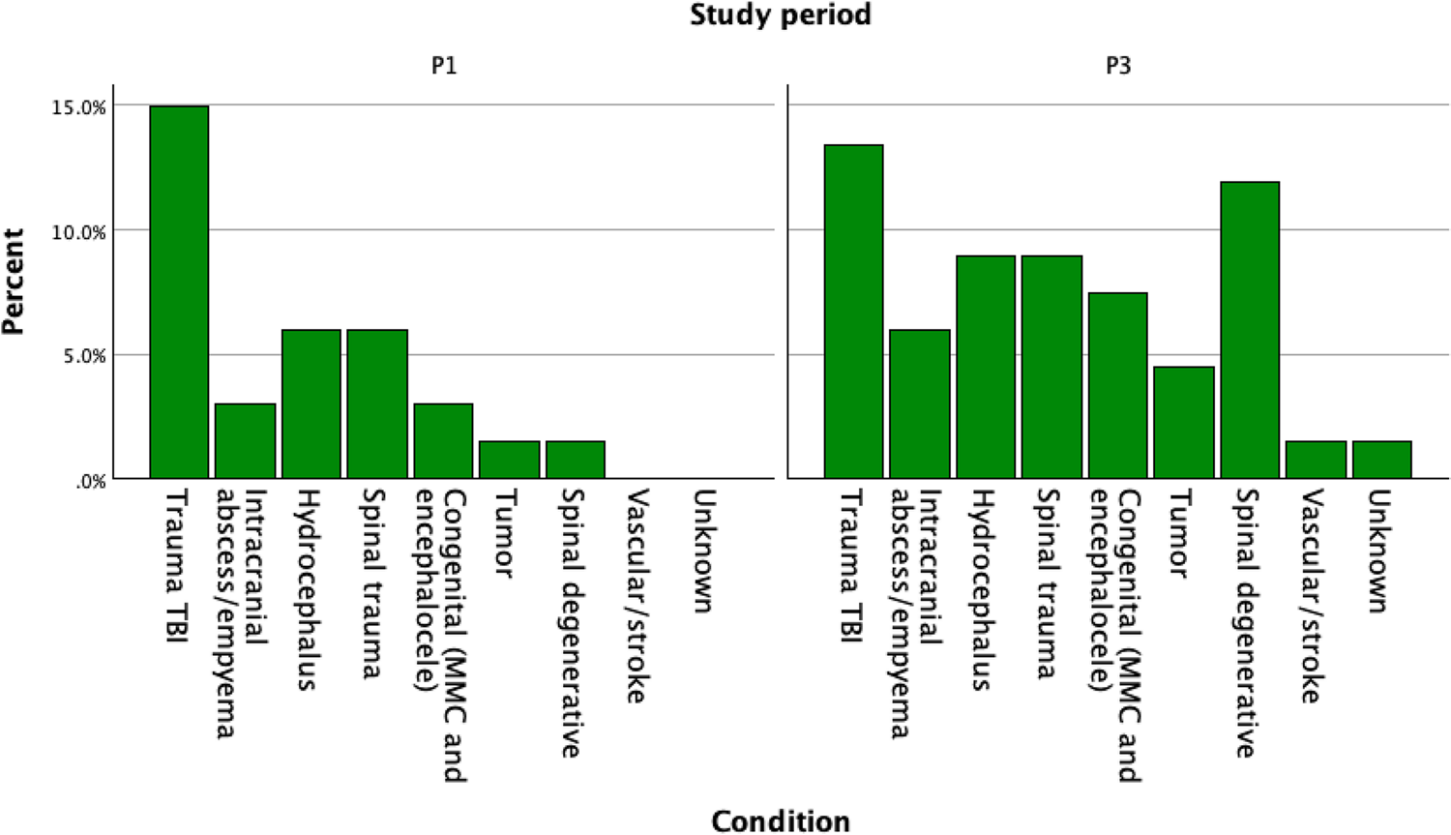
Overview of study period 1 (P1, prior to mission) and period 3 (P3, after the mission)

During the 3 months following the SANC mission (P3), the number of cases (*n* = 43) was higher than during P1 and P2. The cases consisted of TBIs (*n* = 9), spinal degenerative conditions (*n* = 8), hydrocephalus (*n* = 6), spinal trauma (*n* = 6), congenital conditions (*n* = 5), intracranial infections (*n* = 4), intracranial tumors (*n* = 3), and a ventricular hematoma (*n* = 1). In total, 27.0% of the total 89 cases were performed prior to the mission and 48.3% in the period following it (see Fig. 3). During P3, the caseload was 80% when compared to P1.

Postoperative complications of the cases operated under the mission occurred in two cases; 3-month mortality due to complications from diabetes in one patient undergoing cranioplasty and one patient with neurological deterioration after posterior fixation for Pott’s disease in which revision surgery was needed.

### Peri- and postoperative neurosurgical care and education

The SANC surgical nurses (TR, SR) worked in collaboration with the local colleagues to establish surgery a kit with instruments for cranial and spinal surgery cases. Intraoperative teaching was performed during the surgeries, regarding handling of the instruments, and general routines in the operation theater and to establish the routine use of the WHO checklists prior to and after surgery.

The teaching from the neuroanesthesiologists to local personnel occurred daily. A plethora of topics were covered, including importance of blood pressure, saturation, and ventilation with focus on end-tidal carbon dioxide in the anesthetized neurosurgical patient.

A training program for neurosurgical care and nursing was initiated prior to the mission. Three online lectures were held by the neurointensive care specialist nurses (CS, CF) with Gambian nurses and nursing students. During the first day of the mission, 30 nurses participated in lectures and training before hands-on training started. A dedicated neurosurgical postoperative ward was created, staffed by the mission nurses in collaboration with the nurses undergoing the training program. The ward, with eight beds, cared for all patients operated at Ndemban and advanced neurosurgical care, including safe use of external ventricular drains, was administered. Following the mission, altogether four follow-up lectures on neurosurgical nursing has been held monthly online. The participation has extended beyond The Gambia to nurses from other West African countries, including Ghana, Liberia, and Nigeria.

An occupational therapist (CL) assessed all the patients operated during the mission and four patients at the outpatient clinic, including patients with spinal cord injury and stroke/intracerebral hematoma. Patients with spinal cord injury received both written and verbal information on how to prevent decubitus and how to properly move for those with paraplegia, with a goal of achieve as much independency as possible. Additionally, information regarding website on spinal cord injury was shared (SCIRE Community-Spinal Cord Injury Research). The occupational therapist held a lecture for the 40 members of the physiotherapy department at EFSTH discussing both the psychological and medicinal aspects in the treatment of the disabled patients (the encountering of a patient with a serious motor deficit of different origins and the achieving of independency after a serious disability, prevention of contractures, and decubitus). The same lecture was also presented for the nurses at the recovery ward on the first day of the mission.

### Infrastructure

The case of the essential medical equipment was included in the mission. A biomedical engineer (AE) serviced three mobile x-rays (C-arm) four times, in addition to 15 surveillance monitors, three ventilators, five oxygen regulators with tank, one mammography device at the main hospital, and several other small appliances. AE was involved with the set-up of the operating microscope and remote-viewing function, as well as instruction for future service (change of lamp bulb and balancing). AE was involved in education of technician to perform a complete inventory of appliances. He was also taking part in servicing the operation table, to properly vent the anesthesia gas byproducts, and keeping the doors to the operation theaters closed.

### Donations

Multiple donations were made through the mission participants. The most substantial of the donation was the operating microscope (brand Möller-Wedel), which opened the possibilities with microneurosurgery. Other important appliances included a Sonopet ultrasound aspirator and a bipolar instrument and device set. Numerous donations with consumable material such as dura repair material, dura substitution, tissue glue, gloves, sutures, and so on were given to the local hospital at arrival.

### Logistics

An overview on the mission logistics can be found in Table 2.

**Table 2:** Mission logistics

Resource	Response/action
License for medial practice	Obtained through hospital management
VISA	Not needed for travelers from Scandinavian countries
Distance from airport	30 min drive from Banjul International Airport
Security and safety	By local police
Transport	Two minibuses available for transportation
Shipping of equipment	Through seaport and international airport
Customs assistant	Available
Medical risks including vaccination	Necessary vaccinations required
Healthcare if necessary	Available
Climate	Sahelian climate (long dry season followed by short wet season)
Language	English and local language
Food	Continental and local African cuisine. Food during day provided by the hospital
Clean drinking water	Available
Local hospital administrative support	Available
Space for possible expansion of neurosurgery facility and service	Yes (adjoining operating theatres can be adjusted when needed)
Possibility for other areas of co-operation such as research	Yes
Need for training program for staff	Yes
Need to target donations and surgical missions to specific neurosurgery specialties	Yes

## Discussion

The collaborative model of neurosurgical education and cooperation has been proven successful in East Africa to meet the challenges facing neurosurgical development [1, 4]. Density of neurosurgeons varies between parts of Africa, and neurosurgical training is essential to meet the massive needs [9]. The need of neurosurgical training in West Africa has been highlighted previously, with suggested collaborative efforts to overcome the low number of neurosurgeons in the West Africa region [6].

A successful neurosurgical collaborative mission was performed with The Gambia adopting the previously described INTIMA model [5], supporting the use of this approach in future neurosurgical missions. The most important achievements of the mission were the intensive and effective training of the local personnel, the implementation of an operating microscope, and establishing possibilities for further implementation of microneurosurgical techniques. The operation volume of EFSTH after the mission was almost doubled compared to the same time period before the mission and the complexity (classified according to BUPA) of the cases performed, such as intracranial tumors surgery (CMO 4), degenerative spinal surgery (MAJOR + 4 – CMO 3), and congenital including MMC and encephalocele (CMO 2), increased after the mission period.

The surgical performance is at the core of the neurosurgical care. The implementation of modern neurosurgical care requiring the use of an operating microscope is a key step in the development of the local neurosurgical training and while opening the possibility for a future training center. During this mission, an operating microscope was installed, and microneurosurgical principles were taught at the neurosurgical theater allowing for more complex neurosurgical cases also in the future.

Besides the critical infrastructure, we consider the implemented medical and paramedicinal knowledge the most valuable and effective achievement of the mission. Interprofessional education was performed daily between neurosurgeons, nurses, and anesthesiologists. Besides the surgeons, all members of the multidisciplinary team were included in the local education in neurointensive care nursing and occupational and physiotherapeutic care. The reparation service and education of the local staff by the biomedical engineer was highlighted by the local unit due to its crucial economical profitability. After the mission, the initiated training program for neurosurgical care and nursing has continued to extend beyond The Gambia to other West African countries, including Ghana, Liberia, and Nigeria, proving the vast interest among the nursing personnel. This multinational impact of the mission strengthens the twinning technique. Collaboration over land borders strengths further education and care and allows for adapting to new challenges such as the COVID-19 pandemic [7].

During the mission, a collaborative link with partners from Senegal was initiated. This further strengthens the INTIMA model, as exemplified by the initial successful contacts established before this mission during previous missions and outreaches between JJ and SANC as an organization. Creating a strong local framework for neurosurgical case management and neurosurgical education is paramount and creates an environment where the neurosurgical expertise present in the region can address areas of need in smaller countries with fewer neurosurgical care providers. This is also well exemplified by the continuing online training course for neurosurgical nurses.

The mission to The Gambia resulted in outstanding results and experiences that encourage future missions within SANC. The INTIMA model is a valuable framework for organizations and partnerships in global neurosurgery.

## Conclusion

This study validated the use of the INTIMA model previously described in a mission by Swedish African Neurosurgical Collaboration (SANC). The model is sustainable and produces notable results with an increased number of surgery as well as more complex surgeries performed after the mission ended. The core strength of the model is in the multidisciplinary team securing all the aspects and steps of the neurosurgical care. Installation of an operating microscope opened for further microsurgical possibilities, excelling the neurosurgical care in The Gambia.

## Data Availability

The data and material are available upon request to the corresponding author.
